# Supplementary material for: Production of Aflatoxin B1 by Aspergillus parasiticus Grown on a Novel Meat-Based Media
Source: Toxins (Basel). 2022 Dec 29;15(1):25. doi: 10.3390/toxins15010025 (PMC9866511; doi:10.3390/toxins15010025)
Supplement: Supplementary file 1 [file toxins-15-00025-s001.zip › toxins-2071870-supplementary.pdf]

Supplementary Materials: Production of Aflatoxin B1 by *Aspergillus parasiticus* Grown on a Novel Meat-Based Media

Iva Zahija, Barbka Jeršek, Lea Demšar, Mateja Lušnic Polak and Tomaž Polak

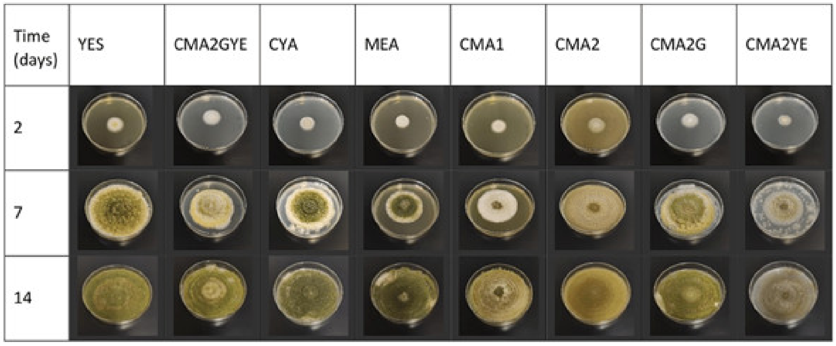

Figure S1. Colonies of *A. parasiticus* after 2, 7 and 14 days of incubation at 25 °C on different media: YES (Yeast Extract Sucrose Agar), CYA (Czapek Yeast Agar), MEA (Malt Extract Agar), CMA1 (Cooked Meat Agar 1), CMA2 (Cooked Meat Agar 2), CMA2G (CMA2 supplemented with 1% of glucose, CMA2YE (CMA2 supplemented with 0.2% of yeast extract, CMA2GYE (CMA2 supplemented with 1% of glucose and 0.2% of yeast extract).

Table S1. Method calibration parameters for different media.

Media	LOQ (pg/column)	LOD (pg/column)	Recovery (%)
CMA1	0.89	0.28	76
CMA2	0.86	0.26	82
CMA2G	0.84	0.25	82
CMA2YE	0.84	0.25	82
CMA2GYE	0.84	0.25	82
YES	0.85	0.26	80
MEA	0.86	0.26	78
CYA	0.89	0.28	76

The limit of quantification (LOQ), limit of detection (LOD) and recovery for YES (yeast extract sucrose agar), MEA (malt extract agar), CYA (Czapek yeast agar), CMA1 (cooked meat agar 1), CMA2 (cooked meat agar 2), CMA2G (CMA2 supplemented with 1% glucose), CMA2YE (CMA2 supplemented with 0.2% yeast extract), and CMA2GYE (CMA2 supplemented with 1% glucose and 0.2% yeast extract).

Table S2. Average production of AFB1 (aflatoxin B1) by *A. parasiticus* grown on different media at 25 °C (ng/mL media).

	Media							
**Day**	**YES**	**CMA2GYE**	**CYA**	**MEA**	**CMA1**	**CMA2**	**CMA2G**	**CMA2YE**
1	18.36 ± 7.05$^{g, 1,2}$	2.25 ± 0.57$^{k, 3}$	13.01 ± 5.80$^{g ,2}$	23.91 ± 20.57$^{f, 1}$	9.43 ± 3.37$^{h, 2,3}$	1.73 ± 0.16$^{i, 3}$	0.00 ± 0.00$^{i, 3}$	0.40 ± 0.28$^{e, 3}$
2	113.22 ± 97.82$^{g, 1}$	10.35 ± 1.97$^{k ,2}$	145.38 ± 59.58$^{g, 1}$	57.70 ± 55.29$^{f, 2}$	22.90 ± 4.44$^{g, 2}$	2.59 ± 1.03$^{i, 2}$	1.44 ± 2.03$^{i, 2}$	3.91 ± 1.21$^{d, 2}$
3	4598.58 ± 706.45$^{f, 1}$	901.51 ± 110.15$^{j,k, 2}$	411.42 ± 86.43$^{g, 3}$	403.98 ± 90.32$^{e, 3}$	23.85 ± 1.33$^{g, 4}$	52.32 ± 17.56$^{h, 4}$	37.28 ± 6.77$^{i, 4}$	5.12 ± 1.26$^{d, 4}$
4	8389.11 ± 2951.38$^{e, 1}$	1609.06 ± 189.93$^{i,j, 2}$	1022.10 ± 52.40$^{f, 2,3}$	1321.88 ± 354.33$^{d, 2,3}$	29.18 ± 7.77$^{f,g, 3}$	64.12 ± 21.38$^{g,h, 3}$	268.37 ± 48.51$^{h, 3}$	4.95 ± 1.17$^{d, 3}$
5	11274.76 ± 2685.42$^{d,e, 1}$	1962.16 ± 128.93$^{i,j, 3}$	1981.53 ± 156.59$^{e, 2}$	1535.21 ± 225.52$^{c,d, 3}$	38.37 ± 2.01$^{e,f, 4}$	70.86 ± 18.92$^{f,g, 4}$	410.92 ± 60.55$^{g,h, 4}$	6.29 ± 0.36$^{d, 4}$
6	14206.26 ± 3420.43$^{d, 1}$	2568.35 ± 420.08$^{h,I, 2}$	2402.56 ± 58.34$^{e, 2}$	1640.04 ± 485.66$^{c, 2,3}$	44.90 ± 2.80$^{e, 4}$	70.42 ± 11.15$^{f,g, 4}$	597.65 ± 64.51$^{g, 3,4}$	11.35 ± 4.87$^{c, 4}$
7	24887.23 ± 4455.71$^{a,b,c, 1}$	3643.13 ± 512.03$^{g,h, 2}$	3210.88 ± 476.60$^{d, 2}$	1640.04 ± 485.66$^{b, 2,3}$	43.76 ± 1.69$^{e, 4}$	72.51 ± 10.44$^{f,g, 4}$	1096.53 ± 192.85$^{f, 3,4}$	16.71 ± 0.97$^{b, 4}$
8	21766.51 ± 2275.78$^{c, 1}$	4640.64 ± 516.38$^{f,g, 2}$	4498.84 ± 308.76$^{c, 2}$	2552.78 ± 50.69$^{b, 3}$	70.02 ± 20.45$^{d, 4}$	74.27 ± 3.42$^{f,g, 4}$	1786.84 ± 380.29$^{d, 3}$	17.17 ± 3.04$^{b, 4}$
9	24048.45 ± 2114.78$^{a,b,c, 1}$	4901.95 ± 434.96$^{e,f, 2}$	4386.45 ± 786.12$^{c, 2}$	3512.57 ± 292.15$^{a, 3}$	76.53 ± 13.15$^{c,d, 5}$	83.11 ± 5.38$^{f,g, 5}$	1794.56 ± 356.19$^{d, 4}$	16.16 ± 2.88$^{b, 5}$
10	23378.92 ± 4803.84$^{b,c, 1}$	6367.52 ± 304.83$^{d, 2}$	5334.94 ± 943.65$^{b, 2,3}$	3649.50 ± 176.16$^{a, 3}$	79.37 ± 7.96$^{c,d, 4}$	84.67 ± 7.63$^{e,f, 4}$	1299.42 ± 115.21$^{e, 4}$	16.97 ± 1.68$^{b, 4}$
11	25197.84 ± 3898.36$^{a,b,c, 1}$	5946.55 ± 776.98$^{d,e, 2}$	5678.52 ± 197.75$^{a,b, 2}$	3667.95 ± 252.05$^{a, 3}$	86.44 ± 11.08$^{b,c, 4}$	101.50 ± 4.93$^{e, 4}$	2213.43 ± 132.77$^{c, 3}$	16.43 ± 1.88$^{b, 4}$
12	27645.57 ± 2401.50$^{a, 1}$	8142.73 ± 1063.08$^{c, 2}$	5820.22 ± 180.99$^{a, 3}$	3670.82 ± 355.33$^{a, 4}$	103.33 ± 18.48$^{a, 6}$	135.12 ± 28.24$^{d, 6}$	2297.79 ± 99.18$^{b,c, 5}$	18.49 ± 3.17$^{b, 6}$
13	26058.49 ± 4320.63$^{a,b, 1}$	9207.11 ± 1000.65$^{c, 2}$	5638.51 ± 153.18$^{a,b, 3}$	3768.08 ± 148,80$^{a, 4}$	104.57 ± 20.44$^{a, 5}$	160.26 ± 16.71$^{c, 5}$	2485.57 ± 183.05$^{b, 4}$	22.94 ± 2.83$^{a, 5}$
14	26139.75 ± 1864.85$^{a,b, 1}$	10929.24 ± 2614.34$^{b, 2}$	5214.00 ± 260.73$^{b, 3}$	3773.57 ± 91.87$^{a, 4}$	94.37 ± 8.35$^{a,b, 5}$	177.79 ± 24.25$^{b, 5}$	2498.19 ± 127.51$^{b, 4}$	16.13 ± 2.46$^{b, 5}$

The content of AFB1 in YES (yeast extract sucrose agar), MEA (malt extract agar), CYA (Czapek yeast agar), CMA1 (cooked meat agar 1), CMA2 (cooked meat agar 2), CMA2G (CMA2 supplemented with 1% glucose), CMA2YE (CMA2 supplemented with 0.2% yeast extract), and CMA2GYE (CMA2 supplemented with 1% glucose and 0.2% yeast extract); a-i data with different superscript letters within column (medium) differ significantly ($p \leq 0.05$); 1-6 data with different superscript numbers within rows (day) differ significantly ($p \leq 0.05$).
